# Compared with the monocyte to high-density lipoprotein ratio (MHR) and the neutrophil to lymphocyte ratio (NLR), the neutrophil to high-density lipoprotein ratio (NHR) is more valuable for assessing the inflammatory process in Parkinson’s disease

**DOI:** 10.1186/s12944-021-01462-4

**Published:** 2021-04-19

**Authors:** Zhu Liu, Qingli Fan, Shizheng Wu, Yaqi Wan, Yancheng Lei

**Affiliations:** 1 Qinghai Provincial People’s Hospital, Xining, China; 2 Qinghai University, Qinghai, China; 3 Key Laboratory of Application and Foundation for High Altitude Medicine Research in Qinghai Province (Qinghai Utah Loint Research Key Lab for High Altitude Medicine), Xining, China

**Keywords:** Parkinson’s disease, Neutrophil to ratio of lipoprotein with high density, Ratio of neutrophil to lymphocyte, Monocyte to high-density lipoprotein ratio, Disease duration, Severity

## Abstract

**Background:**

The inflammatory response plays essential roles in the pathological process and prognosis of Parkinson’s disease (PD). This research investigated the predictive value of the neutrophil to high-density lipoprotein ratio (NHR), neutrophil to lymphocyte ratio (NLR), and monocyte to high-density lipoprotein ratio (MHR) for PD.

**Methods:**

Patients with PD (*n* = 98) were divided into three groups according to disease duration: < 6 years (*n* = 55), 6–10 years (*n* = 29) and > 10 years (*n* = 14). Based on the classification system of Hoehn and Yahr, grades 1 ~ 2.5 were considered early-stage PD (*n* = 44), and grades 3 ~ 5 were considered advanced-stage PD (*n* = 54). In addition, healthy subjects (*n* = 98) matched to the above PD patients in the same period were selected as the control group. Differences in the NHR, NLR, MHR and other indicators among the groups were evaluated.

**Results:**

Smoking, drinking, the neutrophil count and the NHR and NLR were remarkably greater and hypertension, index of body mass, the lymphocyte count, and the levels of cholesterol in total, triglycerides, lipoprotein cholesterol with low density and uric acid were sharply lower in the PD group compared with in the control group. Analysis of multifactor logistic regression indicated that the NHR (odds ratio (adjusted OR) = 1.576, 95% CI: 1.053 ~ 2.358, *P* = 0.027) and NLR (adjusted OR = 1.734, 95% CI: 1.046 ~ 2.876, *P* = 0.033) were factors of risk for PD, while the MHR was not significantly correlated with PD. The areas under the receiver operating characteristic (ROC) curve (AUCs) for the prediction of PD by the NHR and NLR were 0.654 (95% CI: 0.583 ~ 0.721, *P* = 0.0001) and 0.69 (95% CI: 0.62 ~ 0.754, *P* < 0.0001), respectively, and the optimal cutoff values were 1.848 × 10^9^/mmol and 2.62 × 10^9^/mmol. Spearman’s correlation analysis indicated that the NHR was correlated with the disease duration significantly negatively and that the MHR was positively correlated with disease severity.

**Conclusions:**

In summary, the NHR not only has strong predictive value for PD but is also closely related to disease duration. The NHR may be a better prediction for the long-period clinical results in PD patients than the MHR and NLR.

**Trial registration:**

Clinical medical reserach center project of Qinghai Province (2017-SF-L1).

## Background

Parkinson’s disease (PD) is a typical neurodegenerative disease. Its global prevalence rate increases with age. The increase in the prevalence rate is faster than that of other neurological diseases. Ageing of the population is a major driver of the increasing prevalence of PD [1]. Oxidative stress, dyslipidaemia as well as inflammation act key roles within the pathogenesis and progression of PD [2–4]. The proliferation of microglial cells (MGs) and aggregation of a-synuclein (a-syn) mutations within the brain are the pathophysiological bases of PD. MGs secrete high levels of proinflammatory mediators, which damage neurons, further activate MGs, and promote the inflammatory response [5]. In addition, a-syn aggregation stimulates microglial overactivation, which induces a more active inflammatory response [6]. Normally, the blood-brain barrier protects the brain very well. However, since the blood-brain barrier in PD patients is destroyed [7], peripheral inflammation spreads and leads to central inflammation. The levels of neutrophils, monocytes, and lymphocytes, which are white blood cells (WBCs) found in circulating blood, increase when there is peripheral inflammation and participate in the development of clinical symptoms in PD patients [2, 8, 9]. The antioxidant effects as well as anti-inflammatory of high-density lipoprotein cholesterol (HDL-C) are well known, and reduced risks of HDL-C are relevant to systemic inflammation and endothelial cell dysfunction, thus participating in the progression of PD [4, 10]. Studies have found that the decline in the plasma HDL-C level is highly relevant to the high prevalence as well as severity of PD [11, 12].

PD is a heterogeneous disease with slowly and rapidly progressing forms; although some treatments exist (e.g., dopamine agonists, MeDOPA and other pharmacologic treatments, systematic exercise regimens, and rehabilitative therapies), none can reverse the symptoms or prevent the progressive worsening of symptoms, progressive neurodegeneration and the increase in concomitant symptoms. Therefore, PD confers a serious burden on society. Currently, the diagnosis of PD and its differentiation from other neurodegenerative diseases rely mainly on neurological symptoms and psychological cognitive function assessments, magnetic resonance imaging, positron emission tomography (PET) and other methods [13]. In addition, other groups have developed a blood-based classifier that includes Aβ40, total Tau, and a-syn levels to monitor the progression of PD, and α-synuclein levels were shown to have high specificity and accuracy in this effort [14]. However, the relatively high cost of monitoring these levels may limit its large-scale practical application in middle-aged and elderly populations. Previous reports have shown that abnormal lipid metabolism can trigger the aggregation of a-syn [6, 15] and that the aggregation of a-syn can cause the hyperactivity of monocytes in the extracellular fluid, which together activates the response of inflammation of the central nervous system.

In conclusion, we speculated that monocytes, neutrophils, HDL-C and other blood indicators may be effective indicators for the diagnosis and progression of PD in clinical work. Some studies have found that the neutrophil to lymphocyte ratio (NLR), the monocyte to HDL-C ratio (MHR) and the neutrophil to HDL-C ratio (NHR) can be used as potential markers of systemic inflammation, and some studies have found that the NLR is closely related to PD [16–20]. At present, the relationships between the NLR, NHR and MHR and the course and severity of PD are not well understood. Therefore, it is very important to study the correlation of inflammatory factors with PD to facilitate the development of effective preventive and treatment measures for this disease.

## Methods

### Study population

A retrospective analysis was conducted with 98 patients hospitalised in the Department of Neurology of Qinghai Provincial People’s Hospital during March 2017 and March 2020. The study population consisted of 43 females (43.9%) and 55 males (56.1%), ranging from 37 to 86 years old (66.4 ± 12.5 years old). Additionally, sex- and age-matched healthy subjects (*n* = 97) were chosen as the group of control. According to the duration of disease, PD patients were divided into three groups: < 6 years (*n* = 55), 6–10 years (*n* = 29) and > 10 years (*n* = 14). On the basis of the classification system by Hoehn and Yahr, patients were classified as having either early-stage disease (grades 1 ~ 2.5, *n* = 44) or advanced-stage disease (grades 3 ~ 5, *n* = 54). Blood indexes of the healthy control subjects and PD patients were compared according to different age groups (< 50 years old, 50–65 years old, > 65 years old) and sex. The inclusion criteria were as follows: (1) had complete relevant clinical and laboratory examination data and 2) met the diagnostic criteria established by the International Parkinson’s Disease and Movement Disorders Association [21]. The criteria for exclusion were as below: (1) Frontotemporal dementia, Alzheimer’s disease and other neurodegenerative disorders; (2) complications, namely, diabetes, hypothyroidism, tumours and blood immune system diseases; (3) severe liver and kidney injuries; (4) history of stroke or head trauma; and (5) acute infection caused by a fracture, trauma, etc. The research was approved by the ethics committee of Qinghai Provincial People’s Hospital.

### Blood analysis methods

Basic information, such as age, sex, smoking, drinking, anamnesis, height and weight, was recorded. The ratio of weight to the square of height (kg/m^2^) was employed for the calculation of the body mass index (BMI). Medications specific to the treatment before and during hospitalisation were also included. Blood tests were performed on all the subjects immediately after admission. Blood samples were obtained, and the counts of WBCs, neutrophils, monocytes and lymphocytes and the levels of haemoglobin (Hb), HDL-C, LDL-C and uric acid were determined. The MHR, NHR and NLR were calculated. A Beckman AU5800 automatic biochemical analyser was used to measure HDL-C (selective inhibition method), LDL-C (surfactant clearance method) and uric acid (uricase method). The counts of WBCs, neutrophils, monocytes, and lymphocytes; Hb level; and other laboratory indicators were assessed with the Sysmex XN-1000 analyser (Sysmex, Kobe, Japan). The normal plasma concentrations of neutrophils, lymphocytes, and monocytes were 1.8 ~ 6.3, 1.1 ~ 3.2, and 0.1 ~ 0.6 × 10^9^/L, respectively; the normal range of HDL-C was 1.16 ~ 1.42 mmol/L.

### Statistical analyses

SPSS 25.0 software (Chicago, IL, USA) was employed. The normality of the distribution of the measurement data was tested via the Shapiro-Wilk test, and normally distributed variables were shown as the mean ± standard deviation (mean ± SD). A t-test or one-way analysis of variance (ANOVA) was used. Non-normally distributed data are expressed as medians (quartiles). Wilcoxon rank-sum tests were used, and chi-square tests were employed for enumeration data. To study the factors influencing PD, univariate analysis was first conducted, and significant variables (*P* < 0.05) were involved within the multivariate logistic regression model with PD as the dependent variable to determine the factors of risk. Receiver operating characteristic (ROC) curve analysis was employed for the determination of the factors affecting PD and their diagnostic value. Spearman correlation analysis was used. In this analysis, *P* < 0.05 was considered significant statistically.

## Results

### Comparison of baseline demographic features and laboratory indicators of the two groups

In the PD group (*n* = 98), there were 55 males (56.1%) with an age of 68.5 years (59 ~ 77). In the group of control (*n* = 97), there were 52 males (53.1%), with an age of 67.5 years (56.75 ~ 75). There were no obvious differences concerning age as well as sex within the two groups (*P* > 0.05), indicating their comparability. The WBC count, monocyte count, Hb level, and MHR were not changing significantly between the two groups. Smoking, drinking, the use of statin medication, the neutrophil count, and the NHR and NLR were significantly higher, whereas hypertension, the body mass index, the lymphocyte count, and the levels of total cholesterol, triglycerides, HDL-C, uric acid as well as low-density lipoprotein cholesterol (LDL-C) were lower significantly within the group of PD compared with the group of control (*P* < 0.05, *P* < 0.01, Table 1).

**Table 1:** Characteristics of PD patients and healthy controls

	Controls (*n* = 97)	PD patients (*n* = 101)	Z/T	*P*
Sex (male, %)	52 (53.1%)	55 (56.1%)	0.185	0.667
Age (years)	67.5 (56.75 ~ 75)	68.5 (59 ~ 77)	−0.587	0.557
Smoking (male-%)	5 (5.1%)	13 (13.3%)	3.915	0.048
Drinking (male-%)	1 (1%)	8 (8.2%)	–	0.035△
Hypertension (male-%)	32 (32.7%)	19 (19.4%)	4.479	0.034
Statin therapy	13 (13.3%)	27 (27.6%)	6.156	0.013
BMI (kg/m^2^)	24.69 (22.56 ~ 27.39)	23.02 (21.5 ~ 24.63)	−3.812	< 0.001
WBCs (×10^9^/L)	5.53 ± 1.16	5.65 ± 1.12	−0.713	0.477
Neutrophils (×10^9^/L)	3.21 (2.62 ~ 3.88)	3.5 (2.94 ~ 4.37)	−2.646	0.008
Lymphocytes (×10^9^/L)	1.68 (1.32 ~ 2.11)	1.38 (1.15 ~ 1.79)	−3.326	0.001
Monocytes (×10^9^/L)	0.39 (0.32 ~ 0.44)	0.36 (0.31 ~ 0.48)	−0.06	0.952
Hb (×10^9^/L)	161.69 ± 20.26	157.87 ± 13.98	1.539	0.126
UA (μmol/L)	348.85 ± 77.51	303.09 ± 80.16	4.062	< 0.001
TC (mmol/L)	4.97 (4.27 ~ 5.5)	4.09 (3.72 ~ 4.8)	−5.148	< 0.001
TG (mmol/L)	1.47 (1.15 ~ 2.24)	1.18 (0.87 ~ 1.66)	−3.613	< 0.001
HDL-C (mmol/L)	1.32 ± 0.29	1.17 ± 0.29	3.488	0.001
LDL-C (mmol/L)	2.84 ± 0.82	2.45 ± 0.69	3.606	< 0.001
MHR (×10^9^/mmol)	0.3 (0.23 ~ 0.38)	0.33 (0.25 ~ 0.43)	−1.822	0.068
NHR (×10^9^/mmol)	2.48 (1.74 ~ 3.34)	3.18 (2.33 ~ 4.04)	−3.734	< 0.001
NLR (×10^9^/mmol)	2.01 (1.52 ~ 2.54)	2.59 (2.02 ~ 3.18)	−4.599	< 0.001
Parkinson’s drugs				
Compound levodopa (%)	/	77 (78.57)	/	/
DR agonist (%)	/	29 (29.59)	/	/
MAO-B inhibitors (%)	/	28 (28.57)	/	/
COMT inhibitors (%)	/	8 (8.16)	/	/
Anticholinergic drugs (%)	/	8 (8.16)	/	/

**Abbreviations:** *PD* Parkinson’s disease, *BMI* body mass index, *WBC* white blood cell, *UA* uric acid, *TC* total cholesterol, *Hb* haemoglobin, *TG* triglyceride, *HDL-C* high-density lipoprotein cholesterol, *LDL-C* low-density lipoprotein cholesterol, *MHR* monocyte to HDL ratio, *NHR* neutrophil to HDL ratio, *NLR* neutrophil lymphocyte ratio

**△: Fisher’s exact test**

### Risk factors for PD

**Fig. 1:**
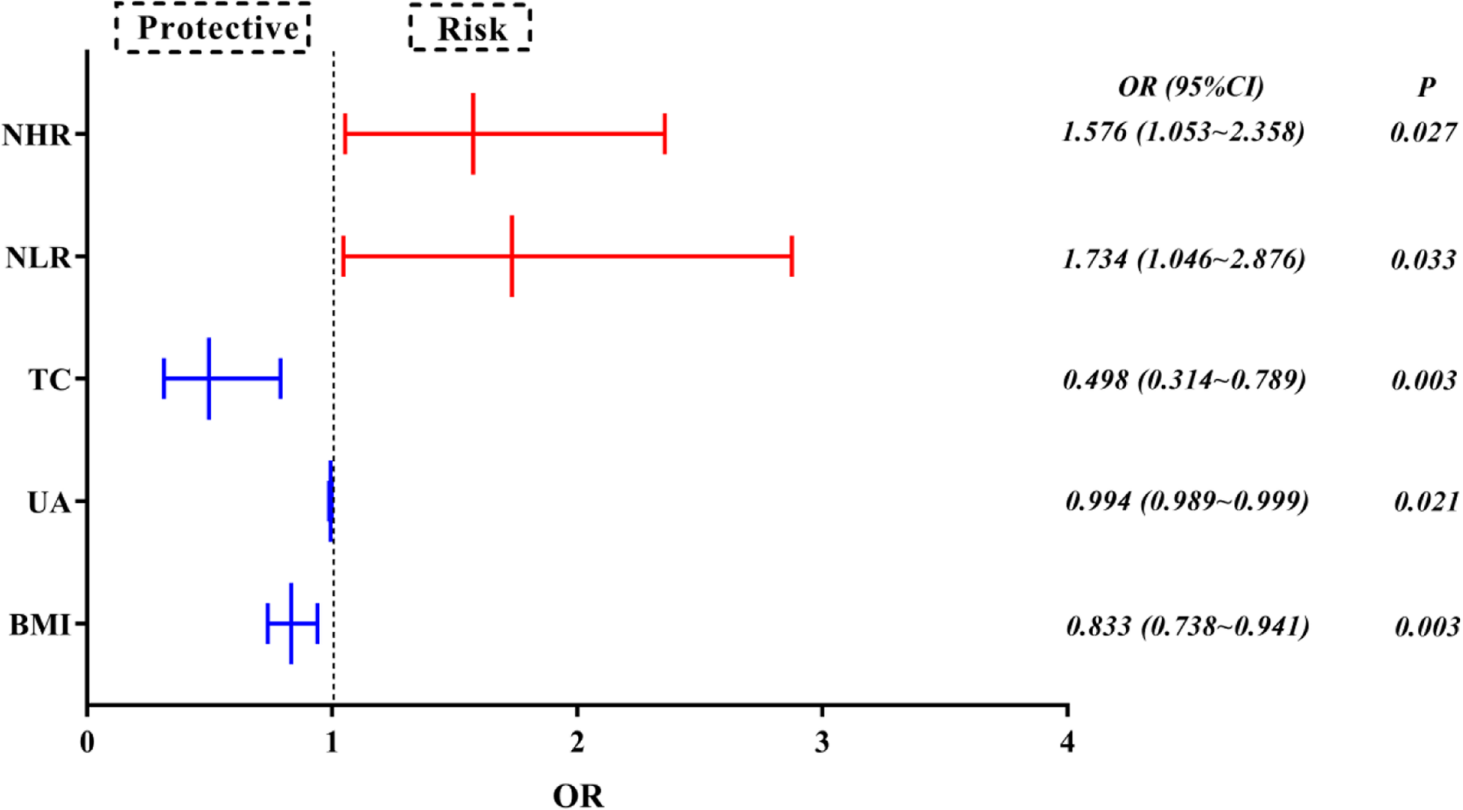
Analysis of risk factors for PD

Univariate analyses indicated that the influencers associated with PD were smoking, drinking, hypertension, the use of statin medication, body mass index, total cholesterol, the levels of uric acid, triglyceride, LDL-C, HDL-C, and the NHR and NLR (because of the collinearity of the NHR and NLR with the neutrophil and lymphocyte counts, the neutrophil and lymphocyte counts were not involved within the multi-factor analysis). To exclude confounding factors such as body mass index, uric acid level, total cholesterol level, triglyceride level, etc., significant factors in univariate logistic analysis were included in the multivariate regression model to obtain an adjusted OR value. The results showed that the above confounding factors did not significantly affect the experimental results. Multi-factor logistic regression analysis illustrated that the NHR (*P* = 0.046, OR = 1.456 > 1) and MHR (*P* = 0.016, OR = 1.663 > 1) were risk factors for PD, while BMI (*P* = 0.003, OR = 0.833 < 1), uric acid level (*P* = 0.021, OR = 0.994 < 1), and total cholesterol level (*P* = 0.003, OR = 0.498 < 1) were protective factors. The results are displayed as a forest plot in Table 2 and Fig. 1.

**Table 2:** Univariate and multivariate logistic analyses of risk factors for PD

	B	Crude OR (95% CI)	***P***	B	Adjusted OR (95% CI)	***P***
Smoking	1.045	2.845 (0.973 ~ 8.314)	0.056	0.42	1.523 (0.394 ~ 5.882)	0.542
Drinking	2.154	8.622 (1.057 ~ 70.309)	0.044	1.704	5.496 (0.446 ~ 67.658)	0.183
Hypertension	−0.701	0.496 (0.258 ~ 0.955)	0.036	−0.318	0.728 (0.31 ~ 1.709)	0.466
Statin therapy	0.911	2.486 (1.195 ~ 5.174)	0.015	0.589	1.803 (0.727 ~ 4.467)	0.203
BMI	−0.192	0.825 (0.749 ~ 0.91)	< 0.001	−0.182	0.833 (0.738 ~ 0.941)	0.003
UA	−0.007	0.993 (0.989 ~ 0.996)	< 0.001	−0.006	0.994 (0.989 ~ 0.999)	0.021
TC	−0.797	0.451 (0.315 ~ 0.644)	< 0.001	−0.698	0.498 (0.314 ~ 0.789)	0.003
TG	− 0.538	0.584 (0.398 ~ 0.857)	0.006	−0.063	0.939 (0.562 ~ 1.57)	0.811
NHR	0.448	1.565 (1.214 ~ 2.018)	0.001	0.455	1.576 (1.053 ~ 2.358)	0.027
NLR	0.873	2.395 (1.616 ~ 3.55)	< 0.001	0.551	1.734 (1.046 ~ 2.876)	0.033

### Subgroup analysis by age

**Fig. 2:**
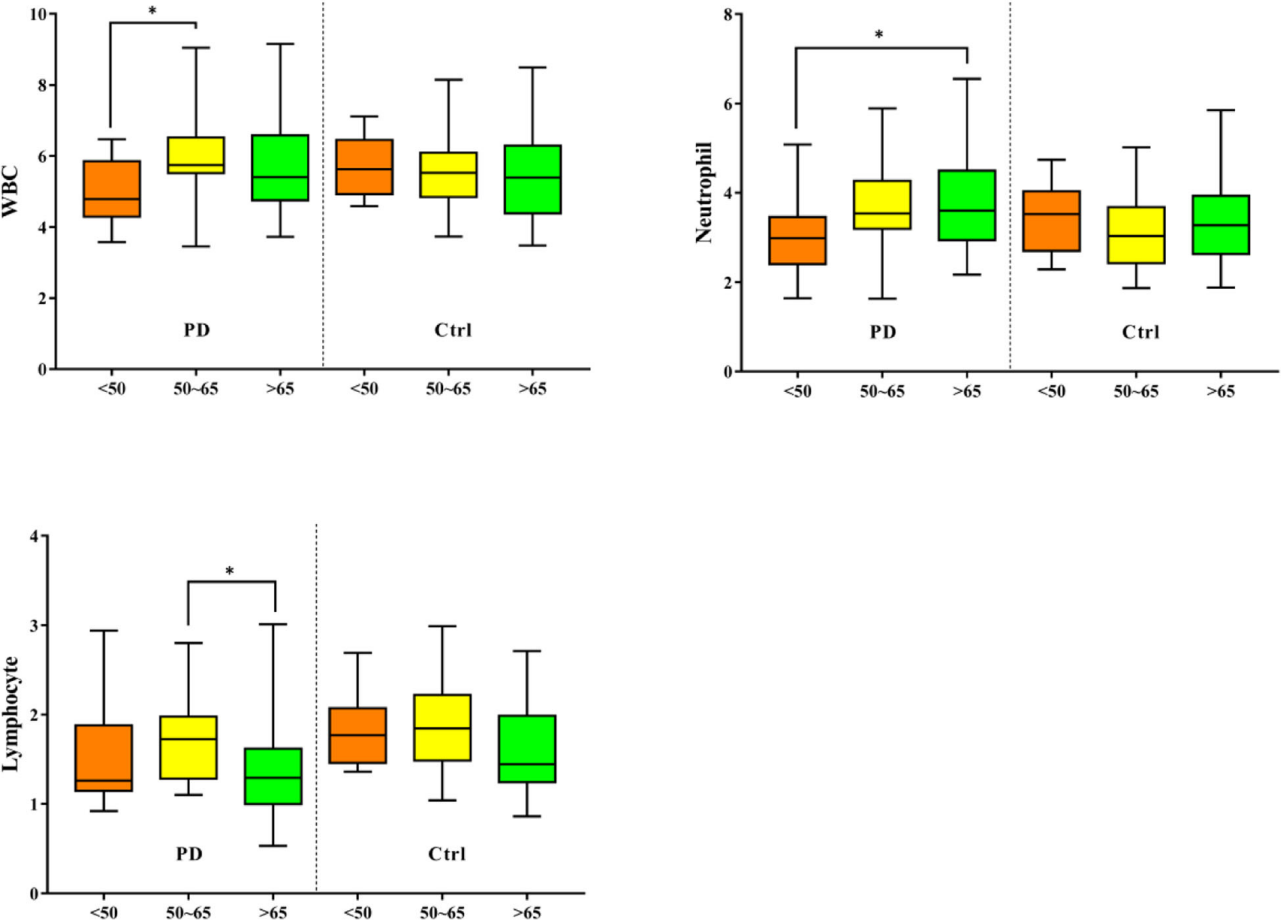
Comparison of the differences between PD patients and healthy controls according to age

There were significant differences in leukocytes, neutrophils, and lymphocytes among different age groups (< 50 years, 50–65 years, as well as > 65 years). Among people < 50 years old, the WBC count of the PD patients was lower significantly compared with that of the healthy controls (4.79(4.29–5.86) vs. 5.63(4.93–6.46), *P* = 0.009). The levels of monocytes Hb, uric acid, cholesterol in total, triglycerides, LDL-C as well as HDL-C were lower in the PD patients compared with in the healthy controls at different ages, but there was no significant difference (Fig. 2).

### Subgroup analysis by sex

**Fig. 3:**
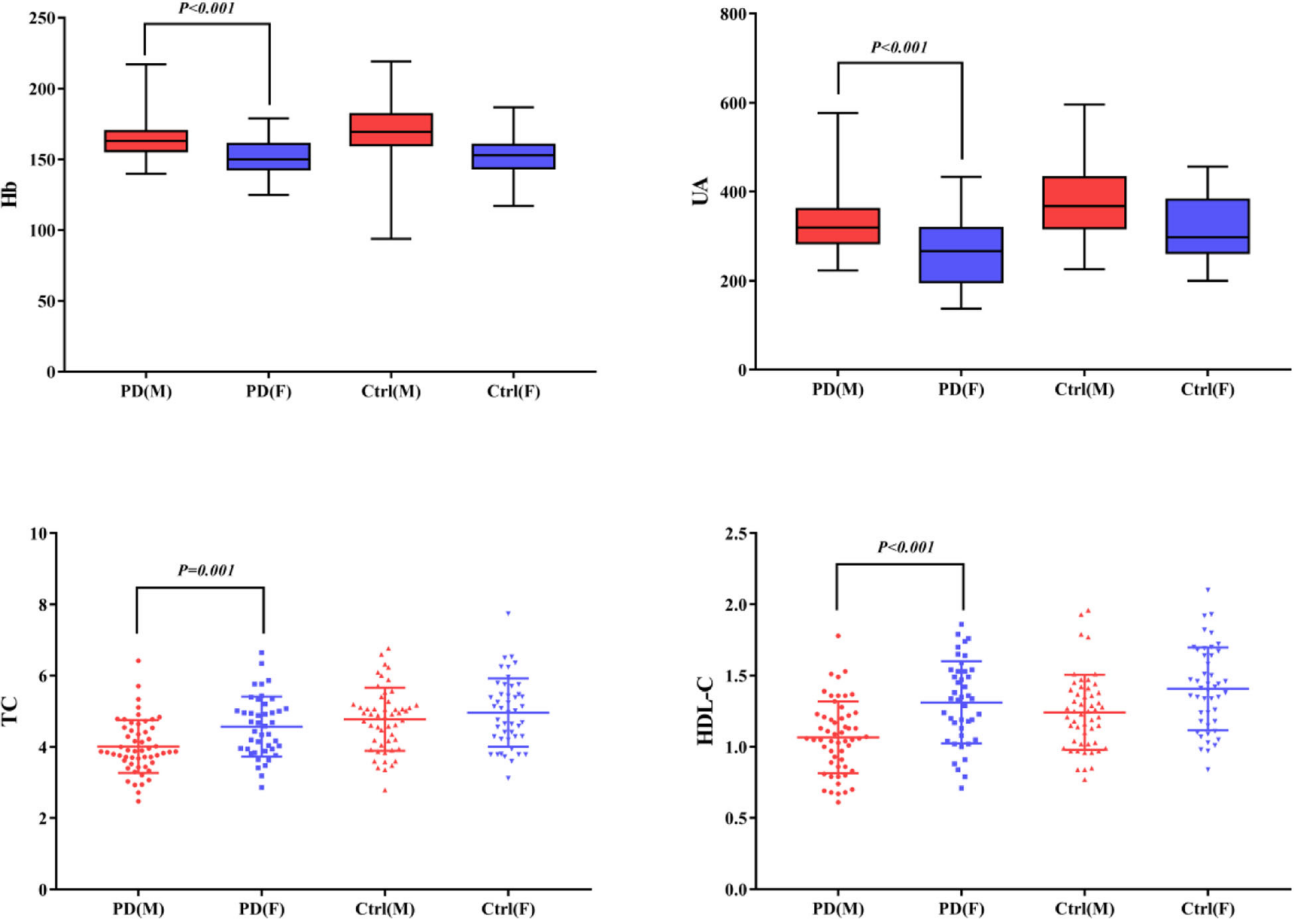
Comparison of the differences between PD patients and healthy controls according to sex

Compared with men, women had significantly higher degrees of Hb, total cholesterol, uric acid, and HDL-C (*P* < 0.05). Within both the patients of PD and healthy controls, the counts of WBCs, neutrophils, and monocytes were greater within men compared with in women, while the lymphocyte count was the opposite (Fig. 3).

### Analysis stratified by disease duration

Pairwise comparisons were carried out among 3 subgroups of patients with disease durations < 6 years, 6–10 years as well as > 10 years. The outcomes showed that the monocyte count was significantly lower in the subgroup of patients with a disease duration 6–10 years than in those with a disease duration < 6 years (*P* < 0.05). The counts of WBCs and neutrophils and the NHR were greater in the patients of PD in the < 6 years groups than those in the > 10 years group. Meanwhile, the levels of WBCs and the MHR and NHR in the group of > 10 years were remarkably lower compared with those in the group of 6–10 years (Table 3).

**Table 3:** Comparison of blood indexes in PD patients with different disease durations

	< 6 years	6 ~ 10 years	> 10 years	χ^2^/F	*P*
N (%)	55 (56.1)	29 (29.6)	14 (14.3)	/	/
WBCs (×10^9^/L)	5.76 (5.08 ~ 6.61)^**b**^	5.53 (4.77 ~ 6.31)^**c**^	4.55 (4.09 ~ 5.6)	11.237	0.004
Neutrophils (× 10^9^/L)	3.61 (3.1 ~ 4.51)^**b**^	3.52 (2.92 ~ 4.45)	2.79 (2.61 ~ 3.42)	7.963	0.019
Lymphocytes (×10^9^/L)	1.54 ± 0.47	1.51 ± 0.62	1.24 ± 0.27	2.129	0.125
Monocytes (×10^9^/L)	0.38 ± 0.1^**a**^	0.44 ± 0.13^**c**^	0.35 ± 0.07	4.072	0.02
Hb (×10^9^/L)	159 (150 ~ 166)	160 (154.5 ~ 165)	150.5 (141.5 ~ 165)	4.503	0.105
UA (μmol/L)	303 (224 ~ 359)	294 (266 ~ 334)	289 (268 ~ 349.5)	0.034	0.983
TC (mmol/L)	4.42 ± 0.8	3.96 ± 0.89	4.25 ± 0.72	3.041	0.052
TG (mmol/L)	1.25 (0.94 ~ 1.83)	1.02 (0.8 ~ 1.61)	1.03 (0.53 ~ 1.18)	5.557	0.062
HDL-C (mmol/L)	1.17 ± 0.29	1.13 ± 0.28	1.3 ± 0.3	1.699	0.188
LDL-C (mmol/L)	2.53 ± 0.73	2.31 ± 0.68	2.43 ± 0.5	0.99	0.375
MHR (×10^9^/mmol)	0.33 (0.25 ~ 0.41)	0.41 (0.26 ~ 0.51)^**c**^	0.27 (0.25 ~ 0.32)	7.477	0.024
NHR (×10^9^/mmol)	3.3 (2.45 ~ 4.14)^**b**^	3.26 (2.41 ~ 4.61)^**c**^	2.07 (1.96 ~ 2.96)	9.975	0.007
NLR (×10^9^/mmol)	2.56 (2.01 ~ 3.41)	2.58 (1.97 ~ 3.39)	2.69 (2.11 ~ 2.87)	0.085	0.958

^**a**^**< 6 years vs. 6 ~ 10 years,** ***P*** **< 0.05; b: < 6 years vs. > 10 years,** ***P*** **< 0.05; c: 6 ~ 10 years vs. > 10 years,** ***P*** **< 0.05**

### Analysis stratified by PD stage

There were no significant variations statistically within the counts of WBCs, neutrophils, lymphocytes, as well as monocytes, in the degrees of uric acid, Hb, cholesterol in total, triglycerides as well as LDL-C, or in the NLR for patients with early-stage as well as middle-advanced-stage PD. However, there were remarkable changes within the HDL-C level, the MHR as well as the NHR (*P* < 0.05). The MHR and the NHR were obviously lower within early-stage PD patients compared with in patients of advanced-stage PD. The HDL-C degree was remarkably greater within early-stage PD patients compared with in advanced-stage PD patients (Table 4).

**Table 4:** Comparison of blood indexes in PD patients with early-stage PD and those with advanced-stage PD

	Early PD	Middle-advanced PD	T/Z	*P*
N (%)	44 (44.9)	54 (55.1)	/	/
WBCs (× 10^9^/L)	5.62 ± 1.26	5.67 ± 1	−0.18	0.857
Neutrophils (× 10^9^/L)	3.58 ± 0.94	3.71 ± 0.96	−0.658	0.512
Lymphocytes (×10^9^/L)	1.5 (1.19 ~ 1.91)	1.3 (1.14 ~ 1.74)	−1.536	0.125
Monocytes (×10^9^/L)	0.38 ± 0.1	0.41 ± 0.12	−1.293	0.199
Hb (×10^9^/L)	157.5 (145.25 ~ 165.75)	159.5 (152 ~ 165)	−0.736	0.462
UA (μmol/L)	299.64 ± 84.77	305.91 ± 76.9	−0.383	0.702
TC (mmol/L)	4.2 (3.78 ~ 4.83)	4.01 (3.51 ~ 4.82)	−0.779	0.436
TG (mmol/L)	1.18 (0.92 ~ 1.47)	1.19 (0.84 ~ 1.67)	−0.146	0.884
HDL-C (mmol/L)	1.27 ± 0.27	1.1 ± 0.29	3.056	0.003
LDL-C (mmol/L)	2.48 ± 0.68	2.44 ± 0.7	0.291	0.772
MHR (×10^9^/mmol)	0.27 (0.23 ~ 0.4)	0.35 (0.28 ~ 0.49)	−2.843	0.004
NHR (×10^9^/mmol)	2.94 ± 0.99	3.65 ± 1.4	−2.903	0.005
NLR (×10^9^/mmol)	2.52 (1.92 ~ 3.01)	2.69 (2.06 ~ 3.36)	−1.071	0.284

### Analysis of ROC curve

**Fig. 4:**
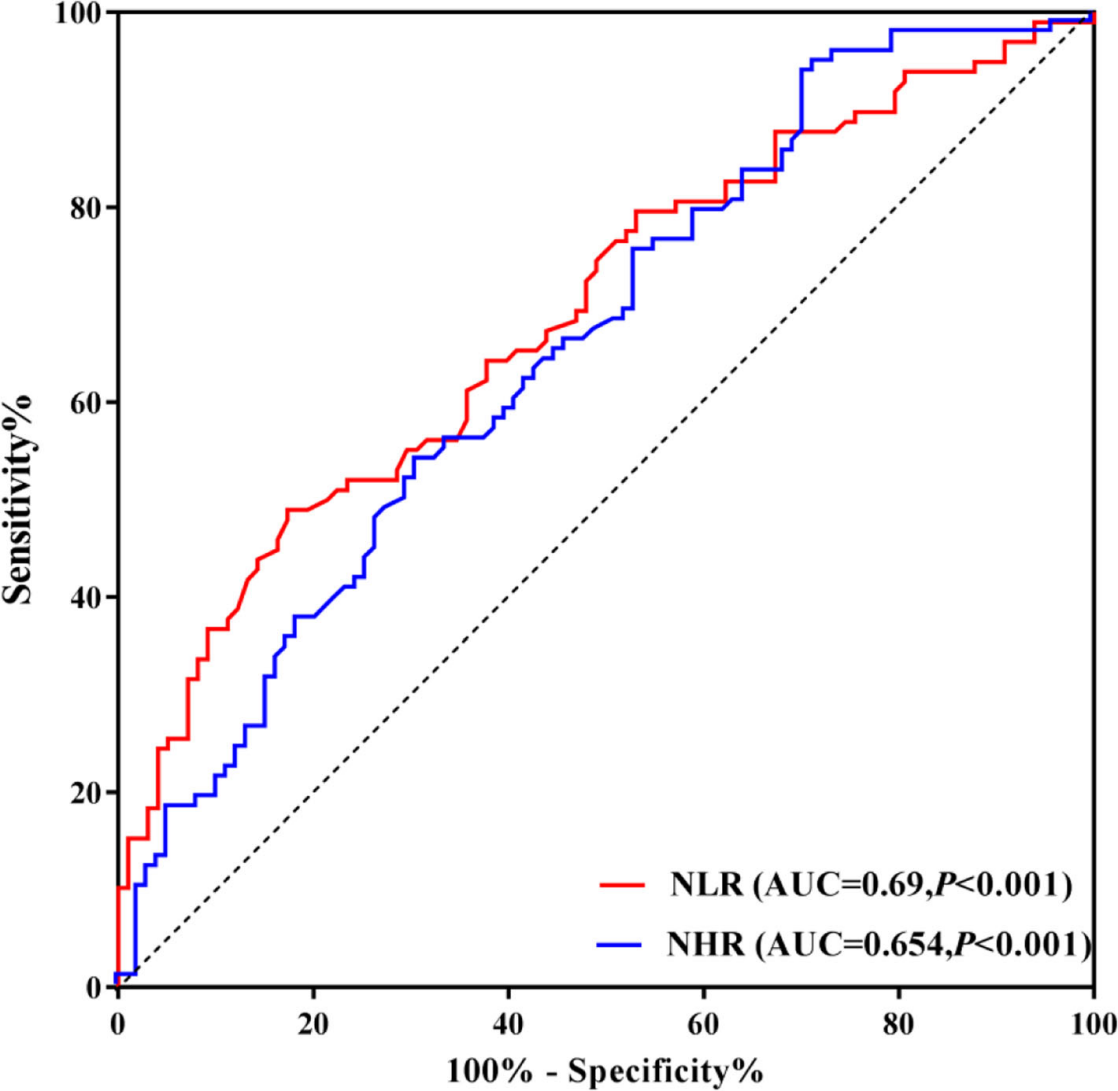
ROC curve analysis of the prediction of PD based on the NHR and NLR

Analysis of ROC curve was conducted to assess the predictive value of the NHR as well as NLR for PD. For the NHR, the AUC was 0.654 (95% CI: 0.583 ~ 0.721, *P* = 0.0001), the best cut-off value was 1.848 × 10^9^/mmol, the sensitivity was 28.57%, and the specificity was 94.9%. The AUC for the NLR was 0.69 (95% CI: 0.62 ~ 0.754, *P* < 0.0001), and its optimal cut-off value was 2.62 × 10^9^/mmol, yielding a specificity of 82.65% as well as a sensitivity of 48.98% (Fig. 4).

### Spearman correlation analysis

Because there were significant differences in the NHR and MHR based on the disease duration and PD stage, a correlation analysis was performed. The outcomes indicated that there was a remarkable negative connection between the NHR and the disease duration, with a correlation coefficient of − 0.223 (*P* < 0.05); there was no remarkable connection between the Hoehn_Yahr classification (*P* > 0.05). There was no significant correlation of MHR with disease duration, and there was a significant positive connection of the Hoehn_Yahr classification and the MHR, with a correlation coefficient of 0.247 (Table 5).

**Table 5:** Correlation analyses of the NHR and MHR with disease duration and PD stage

	NHR	MHR	Disease duration	Hoehn-Yahr
NHR	1			
MHR	.660^b^	1		
Disease duration	−.223^a^	−0.003	1	
Hoehn-Yahr	0.177	.247^a^	.373^b^	1

^a^At the level of 0.05 (two-tailed), there was a significant correlation

^b^At the level of 0.01 (two-tailed), there was a significant correlation

## Discussion

The relationships of the NHR, NLR and NHR with PD duration and stage were investigated. The main findings are as follows: (1) the NHR and NLR are significantly correlated with PD; (2) as the disease progresses, the NHR significantly decreases, and in patients with more advanced PD, the NHR and MHR are higher; however, the NLR is not associated with either disease duration or stage; (3) analysis of the ROC curve indicated that the AUC of the NLR was greater than that of the NHR; and (4) in the Spearman correlation analysis, the NHR was correlated negatively with the duration of disease, as well as the NHR was correlated positively with disease severity. Based on the above results, the NLR has a higher predictive value for PD, while the NHR may be associated with chronic inflammation in PD patients.

A wonderful systematic review as well as meta-analysis of human transcriptomic data was recently performed by Noori et al. [22]. The authors identified neuroinflammation, altered energy metabolism, and defective proteostasis as being common to multiple neurodegenerative diseases. In pan-neurodegenerative diseases, the upregulation of genes involved in enhanced movement and the phagocytosis of microglia and astrocytes leads to neuroinflammation, while the downregulation of genes associated with the electron chain transport of mitochondria and the cytoskeleton drives neurodegeneration. Furthermore, microglia contribute to the secretion of reactive oxygen species (ROS) causing oxidative stress as well as persistent inflammatory responses [23]. The formation of Louis nucleosomes is a characteristic pathological change in PD patients; these nucleosomes are composed mainly of -synuclein (−SYn). An elevated plasma a-syn level is considered an effective biomarker for the diagnosis, differentiation and progression of PD and acts an essential role in the inflammatory activation process of PD in coordination with microglia [6, 15]. Previous studies have shown that neutrophils have a strong ability to pass through blood vessel walls and epithelial cell layers; they recruit, activate, and regulate the transport of different leukocyte populations in tissues, and they promote the body’s inflammatory response through the regulation of chemokines [24, 25]. Our study showed that the neutrophil count was greater significantly within the PD patients compared with in the healthy control subjects, which confirmed the involvement of neutrophils in the onset of PD to a certain extent. Additionally, the neutrophil count was closely related to the HDL-C level. HDL-C can inhibit not only the expression of neutrophil activators (PMA and fMLP) but also neutrophil activation, adhesion, diffusion and migration [26]. This is consistent with the findings of this study, which showed that the neutrophil count decreased as the disease progressed. A meta-analysis [27] illustrated that non-steroidal anti-inflammatory drugs (NSAIDs) are efficient for the treatment as well as prevention of PD and may also lead to a decrease in the neutrophil count. An immunomagnetic reduction method (IMR) was used to detect the dynamic changes in blood biomarkers [14], and it was found that plasma α-synuclein in PD patients was greater significantly compared with that in the healthy group of control and was the highest in Parkinson’s disease dementia (PDD) patients. Moreover, monocytes were found to be closely related to α-SYN in the course of PD. Existing studies have shown [28] that the level of a-syn in the blood of PD patients with the treatment of replacement therapy of dopamine, especially agonists of dopamine, remained relatively low for 12 months after treatment. Although most PD patients received dopaminergic medications in this study, the level of plasma monocytes may have been affected during early treatment. The increased level of monocytes has been suggested to be a manifestation of the severity of PD disease. In an animal experiment, T cell infiltration (e.g., infiltration by CD3^+^ and CD4+ T cells) was detected in the hippocampus, neocortex, as well as striatum perivasculature region and parenchyma of -SYN mice. Immunoregulatory mediators and/or a lack of effective mechanisms of dissolution lead to a pro-inflammatory environment. In addition, T cell interactions with glial cells further enhance inflammatory activity [5, 29]. The outcomes of this research indicated that the lymphocyte count in peripheral blood was significantly lower in the PD patients compared with in the control group, which might be relevant towards the lymphocytes being recruited to the brain parenchyma due to blood-brain barrier dysfunction, leading to an imbalance in lymphocytes and reductions in the CD3^+^ as well as CD4^+^ lymphocyte subsets within patients with PD; this reduction is especially prominent in the subpopulation of CD3^+^ lymphocytes, which is typically the most abundant [7, 30]. In addition, ageing can cause mitochondrial dysfunction and loss of dopaminergic substantia nigra neurons leading to oxidative damage [31]. In this study, with an increase in age, the levels of plasma leukocytes, neutrophils and lymphocytes all showed significant changes. However, the relatively low WBC count in the group < 50 years old was even lower compared with that in the corresponding healthy group of control, which was considered to be related to the compensatory mechanism of PD triggered by oxidative damage. For example, microglia can repair neurons through lipid metabolism and can transform a pro-inflammatory environment into an anti-inflammatory environment [32, 33].

In addition to the relationships between inflammation and PD onset and progression [4, 5, 8], PD is strongly associated with abnormal lipid metabolism [34]. Since lipid metabolism has increased demands during neuronal repair and remodelling, higher lipid levels promote the recovery of neuronal function in PD patients and thus reduce the risk of future disease. In addition, dyslipidaemia is considered an early change in motor symptoms in PD patients [32]. Plenty of reports have indicated that increased degrees of cholesterol in total, triglycerides, HDL-C and LDL-C were negatively correlated with the incidence of PD [11, 12, 32]. HDL-C (especially below 40 mg/dl) appears to affect Parkinson’s etiopathogenesis [11]. On contrary, the relationship between the level of HDL-C in the peripheral blood and PD has not been examined. In existed researches, the PD patients had significantly decreased [35, 36] or unchanged [8, 37, 38] plasma HDL-C levels compared with the group of control. This study found that the level of HDL-C increased gradually with an increasing PD duration, which was consistent with previous studies [39]. Possible explanations include the following: (1) Smoking has been shown to be correlated negatively with the prevalence of PD, and smoking cessation in PD patients may lead to a reduced risk of cardiovascular disease [40]. Additionally, one study demonstrated that smokers had lower HDL-C levels than non-smokers [11]. (2) Levodopa is the standard therapy for PD. Studies have shown that levodopa can reduce blood lipid levels, but as the disease progresses, it is often necessary to reduce the dose of levodopa to avoid motor complications [41]. (3) Other reports have shown that PD patients experience a cardiometabolic protective effect [42]. HDL-C has been recognised as a protective influencer against cardiovascular disease, as well as levels of HDL-C have been found to increase as PD progresses. Surprisingly, this research indicated that the HDL-C levels were decreased significantly within patients with PD of advanced stage compared with within patients with PD of early stage. Normal lipid management is important to sustain microglia metabolism as well as homeostasis. However, dyslipidaemia can cause impaired metabolism, migration and phagocytosis in microglial cells, resulting in the excessive secretion of oxidation products and activation of the inflammatory activation, which is relevant closely towards the loss of PD neurons and the characteristics of movement disorders, leading to the continuous progression of the disease [33]. ApoA1 exists in the cerebrospinal fluid and is the most important structural protein component of HDL-C. Recent studies have demonstrated that high ApoA1 levels can effectively delay dopaminergic neurodegeneration in PD patients, thus reducing disease severity [43]. Since the level of HDL-C varies with age and sex, a subgroup analysis of HDL-C levels and blood indicators was conducted in this study. The outcomes illustrated that the TC and HDL-C levels in women were greater significantly compared with those within men, both in the healthy control group and in the PD patients, which may be related to the higher prevalence of PD in men. Although not statistically significant, levels of HDL-C were likely to decrease with age in PD patients. This is consistent with previous findings that personalles with greater degrees of HDL-C were likely to be female, younger, and have higher TC levels [11, 44]. Large changes in HDL-C were thought to be associated with PD progression, but the effect was not significant based on age or sex [11]. Although lipid levels in this study may have been affected by lipid-lowering therapy with statins, it is known that statins have a small effect on HDL-C. Therefore, the NHR and MHR are relatively accurate in assessing the incidence and severity of PD. HDL-C has long been regarded as the “perfect cholesterol” and “the higher, the better.” Treatments that enhance the function of HDL-C are beneficial for both the peripheral and central nervous systems [43]. However, HDL-C levels above 2.33 mmol/L are relevant to an rised risk of cardiovascular disease according to the 2019 European Society Guidelines for the Management of Dyslipidaemia [45]. Another study found that the notion that higher HDL-C levels are better does not hold true for younger people and men. Degrees of HDL-C in the scale of 1.55 to 2.32 mmol/L in males and 1.81 to 2.32 mmol/L in females might be related to greater mortality [44]. Therefore, it is particularly essential for attention towards the level and dynamic changes in HDL-C in PD patients.

In conclusion, the immune inflammatory reaction and dyslipidaemia are important factors of promotion of the onset as well as progression of PD. The NHR and NLR are the ratios of neutrophils to HDL-C and lymphocytes, respectively, which was the basis for the assumption that these two indexes would be related to PD. They are not only simple and fast to calculate but also integrate multiple factors. Compared with a single indicator, they have the advantage of reflecting the complementary relationships between different pathways, enabling them to more accurately portray the inflammatory changes in PD. Therefore, the NHR and NLR are believed to be closely related to inflammatory activity and changes in lipid profiles. Previous studies have demonstrated that the interaction between an increased neutrophil count and a decreased HDL-C level leads to an increased NHR and an rised risk of accurate myocardial infarction in the elderly population [46]. In an analysis of the NHR and prognosis given intravenous thrombolysis within acute ischaemic stroke patients [17], the NHR increased with the increasing severity of nerve injury, and 25% of the patients with high NHRs developed severe neurological impairment. As a chronic neurodegenerative disease, PD has the greatest neurological burden in patients 80 to 89 years old [1]. Our results showed that the NHR decreased significantly as the disease progressed, and the NHR was decreased within patients with PD of advanced stage compared with in those with PD of early stage. The NHR is negatively correlated with disease duration and can be an efficient predictor of long-period clinical outcomes. The MHR as well as NLR are considered indexes reflecting oxidative stress as well as inflammatory response, and the NLR values within patients with PD are greater significantly compared with those in healthy control subjects, but there is no difference among different clinical subtypes; notably, the conclusions of this study are consistent with these findings [18–20]. Although a decline concerning the number of CD4+ T-cell subsets in PD patients indicates increased systemic involvement [47], a significant increase in lymphocyte apoptosis may affect the NLR to some extent. Our study showed that both the NHR and NLR are useful predictors of PD, but the latter is better. In conclusion, use of the NHR and NLR can improve the diagnosis, treatment and prognostic prediction of PD in clinical practice.

### Study strength and limitations

The negative correlation between NHR and disease duration proves that NHR can be used as a good indicator for predicting the long-term clinical course of PD, which is the strength of this study. However, some limitations were also reported. First, due to the retrospective feature of the research, the effect of the NHR and NLR on the prognosis of PD could not be assessed. Second, the size of the sample was relatively small, as the patients were treated in the same hospital, the generalisability of the results are limited. Finally, the specific mechanisms by which the NHR and NLR affect the onset and progression of PD are still unknown, and more studies are needed.

## Conclusions

In conclusion, lipid metabolism, neutrophils, lymphocytes and monocytes all play an essential role within the emergence as well as progression of PD. This research also supports the close connection between oxidative stress, lipid metabolism as well as inflammatory response and PD. In addition, the NHR may be an effective biomarker of PD and could be used to predict the incidence and severity of PD; this biomarker also has great clinical significance for the diagnosis and prevention of PD.

## Data Availability

The datasets employed and/or analysed within the current research can be accessed from the corresponding authors upon reasonable requests.

## Abbreviations

PD: Parkinson’s disease
BMI: Body mass index
WBC: White blood cell
UA: Uric acid
TC: Total cholesterol
Hb: Haemoglobin
TG: Triglyceride
HDL-C: High-density lipoprotein cholesterol
LDL-C: Low-density lipoprotein cholesterol
MHR: Monocyte to HDL ratio
NHR: Neutrophil to HDL ratio
NLR: Neutrophil lymphocyte ratio
OR: Odds ratio
MGs: Microglial cells
a-syn: A-synuclein
PET: Positron emission tomography
ANOVA: A t-test or one-way analysis of variance
ROC: Receiver operating characteristic
NSAIDs: Non-steroidal anti-inflammatory drugs
IMR: Immunomagnetic reduction method
PDD: Parkinson’s disease dementia
